# Circulating MACC1 Transcripts in Glioblastoma Patients Predict Prognosis and Treatment Response

**DOI:** 10.3390/cancers11060825

**Published:** 2019-06-13

**Authors:** Carsten Hagemann, Nikolas Neuhaus, Mathias Dahlmann, Almuth F. Kessler, Dennis Kobelt, Pia Herrmann, Matthias Eyrich, Benjamin Freitag, Thomas Linsenmann, Camelia M. Monoranu, Ralf-Ingo Ernestus, Mario Löhr, Ulrike Stein

**Affiliations:** 1 Tumorbiology Laboratory, Department of Neurosurgery, University of Würzburg, Josef-Schneider-Str. 11, D-97080 Würzburg, Germany; niko.neuhaus@gmail.com (N.N.); Kessler_A1@ukw.de (A.F.K.); Linsenmann_T@ukw.de (T.L.); Ernestus_R@ukw.de (R.-I.E.); Loehr_M1@ukw.de (M.L.); 2 Experimental and Clinical Research Center, Charité Universitätsmedizin Berlin and Max-Delbrück-Center for Molecular Medicine in the Helmholtz Association, Robert-Rössle-Straße 10, D-13125 Berlin, Germany; dahlmann@mdc-berlin.de (M.D.); Dennis.Kobelt@mdc-berlin.de (D.K.); pia.herrmann@charite.de (P.H.); 3 German Cancer Consortium (DKTK), Im Neuenheimer Feld 280, D-69120 Heidelberg, Germany; 4 Department of Pediatric Hematology/Oncology, University Children’s Hospital, University of Würzburg, D-97080 Würzburg, Germany; Eyrich_M@ukw.de (M.E.); E_Freitag_B@ukw.de (B.F.); 5 Department of Neuropathology, Institute of Pathology, University of Würzburg, Josef-Schneider-Str. 2, D-97080 Würzburg, Germany; camelia-maria.monoranu@mail.uni-wuerzburg.de

**Keywords:** metastasis-associated in colon cancer 1 (MACC1), glioblastoma multiforme, liquid biopsy, therapy response, prognostic marker

## Abstract

Glioblastoma multiforme is the most aggressive primary brain tumor of adults, but lacks reliable and liquid biomarkers. We evaluated circulating plasma transcripts of metastasis-associated in colon cancer-1 (MACC1), a prognostic biomarker for solid cancer entities, for prediction of clinical outcome and therapy response in glioblastomas. MACC1 transcripts were significantly higher in patients compared to controls. Low MACC1 levels clustered together with other prognostically favorable markers. It was associated with patients’ prognosis in conjunction with the isocitrate dehydrogenase (IDH) mutation status: IDH1 R132H mutation and low MACC1 was most favorable (median overall survival (OS) not yet reached), IDH1 wildtype and high MACC1 was worst (median OS 8.1 months), while IDH1 wildtype and low MACC1 was intermediate (median OS 9.1 months). No patients displayed IDH1 R132H mutation and high MACC1. Patients with low MACC1 levels receiving standard therapy survived longer (median OS 22.6 months) than patients with high MACC1 levels (median OS 8.1 months). Patients not receiving the standard regimen showed the worst prognosis, independent of MACC1 levels (low: 6.8 months, high: 4.4 months). Addition of circulating MACC1 transcript levels to the existing prognostic workup may improve the accuracy of outcome prediction and help define more precise risk categories of glioblastoma patients.

## 1. Introduction

Glioblastoma multiforme is the most prevalent, aggressive, invasive, and difficult to treat primary brain tumor of adults. Despite multimodal treatment comprising microsurgical tumor resection followed by local irradiation and systemic temozolomide (TMZ) chemotherapy, the median survival is less than 15 months and relapse is unavoidable [1,2,3]. 

The isocitrate dehydrogenase (IDH) mutation status and methylation of the O-6-methylguanine-DNA methyltransferase (MGMT) gene promoter are established prognostic markers of GBM evaluated in patients’ tumor samples [1,4,5,6,7]. Blood biomarkers of prognostic importance, as established in clinical care of patients with various other cancer types [8], have not been introduced for GBM patients on a routine basis [9].

Metastasis-associated in colon cancer 1 (MACC1) is a prognostic and predictive biomarker for metastasis formation and metastasis-free survival of patients with colon cancer [10] and other solid tumors [11,12]. Circulating MACC1 transcripts are established as prognostic plasma marker for several cancer entities [13,14,15]. We showed that MACC1 overexpression increases the proliferative, migratory, and tumor-formation abilities of GBM cells in vitro and in organotypic hippocampal slice cultures of mice. Increased MACC1 expression in biopsies of GBM patients is associated with reduced patient survival [16]. Here, we analyze whether MACC1 could serve as a prognostic biomarker for GBM patients before initial surgery that can be detected using routine diagnostic liquid biopsies.

We report that MACC1 transcripts are detectable and enhanced in the blood of GBM patients, cluster together with other prognostic markers of clinical importance, and are associated with patients’ prognosis in conjunction with the IDH1 mutation status and the treatment regimen.

## 2. Results

### 2.1. Patient Cohort

Pre-surgical blood specimens of a total of 45 patients with a confirmed GBM were assessed for circulating MACC1 transcripts. Patients’ characteristics, tumor characteristics, details about therapy, and outcome are summarized in Table 1. According to the differential blood count, the immune profile of all patients was normal. In total, 50% of the patients were MGMT promoter methylated. Six patients (13%) possessed an IDH1 R132H mutation (IDH1 MT). Tumors not possessing this mutation are referred to as IDH1 wildtype (IDH1 WT) [6]. The clinical course of all patients was followed for 24 months.

### 2.2. MACC1 Transcript Concentrations Were Higher in the Plasma of GBM Patients

**Figure 1:**
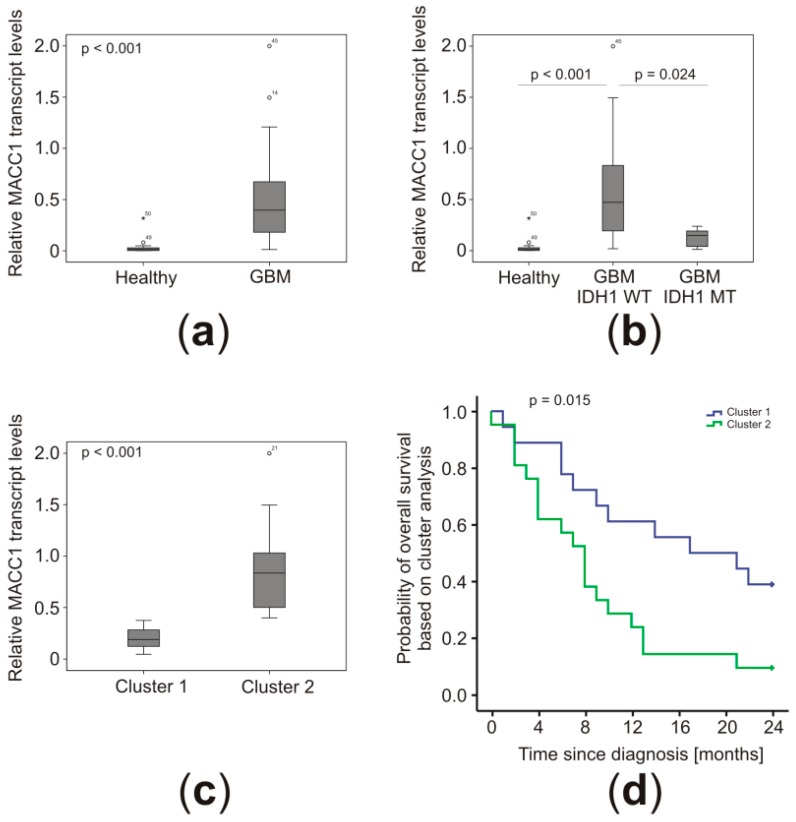
Metastasis-associated in colon cancer-1 (MACC1)-transcript levels in the plasma of GBM patients correlated with disease grade and survival. MACC1 plasma levels were determined by quantitative RT-PCR (in duplicates). (**a**) Comparison of all GBM patients (*n* = 45) with healthy controls (*n* = 15). (**b**) Comparison of GBM without (*n* = 39; IDH1 wildtype (WT)) and with IDH1 R132H mutation (*n* = 6; IDH1 MT) and healthy controls (*n* = 15). (**c**) Expression levels of circulating MACC1 transcripts in patients’ plasma after cluster analysis (Cluster 1: *n* = 18; Cluster 2: *n* = 21, for detailed characteristics, please refer to Table 2). (**d**) Kaplan–Meier plot of the patients’ overall survival (OS) according to cluster membership. Statistical analysis was performed using Mann–Whitney-U test (**a**,**c**), one-way ANOVA with Tukey post-hoc analysis (**b**), and log-rank test (**d**).

Based on the identification of MACC1 transcripts in patients’ blood as diagnostic and prognostic biomarkers [13,14], we quantified MACC1 mRNA levels in plasma samples of 45 GBM patients before surgery. On average, MACC1 mRNA concentrations were increased 13.1-fold compared to healthy controls (*p* < 0.001) (Figure 1a). Interestingly, the MACC1 plasma levels of patients with IDH1 MT in their tumors were enhanced 3.4-fold on average compared to healthy controls, but only 23% of the magnitude of patients with IDH1 WT (*p* = 0.024) (Figure 1b). Therefore, MACC1 mRNA was clearly detectable and enriched in the blood of GBM patients. The high interpatient variability from as low as 0.012% calibrator up to 1.999% calibrator allows us to hypothesize that circulating MACC1 transcripts might be of prognostic value, and we performed cluster analyses to detect correlations with clinical data of known prognostic importance.

### 2.3. Low MACC1 Plasma Levels Clustered together with Other Favorable Markers

Cluster analyses were performed based on patient, histological, and molecular criteria to identify co-clustering parameters. Due to missing data for MGMT promoter methylation in five cases or tumor volume for one patient, a total of six patients had to be excluded. The clustering of 39 patients revealed that patients with low MACC1 plasma levels (0.20% calibrator SD = 0.10 vs. 0.84% calibrator SD = 0.41; *p* < 0.0001) were of younger age (57.0 years SD = 11.9 vs. 69.1 years SD = 9.3; *p* = 0.0010), were IDH1 MT, and had a tendency for smaller tumors (33 cm^3^ SD = 24 vs. 44 cm^3^ SD = 28) (Table 2 and Figure 1c). There was no difference in the MGMT status in both clusters. Importantly, these patients lived longer (16.0 months SD = 8.5 vs. 9.3 months SD = 6.9; *p* = 0.0184) (Figure 1d and Table 2), indicating prognostic importance of MACC1 mRNA plasma levels. Thus, we performed Kaplan–Meier analyses to evaluate the prognostic value of MACC1 on both overall (OS) and progression-free (PFS) survival of GBM patients.

### 2.4. MACC1 Levels Correlated with Patient Prognosis in Conjunction with the IDH1 Mutation Status and Treatment Regimen

**Figure 2:**
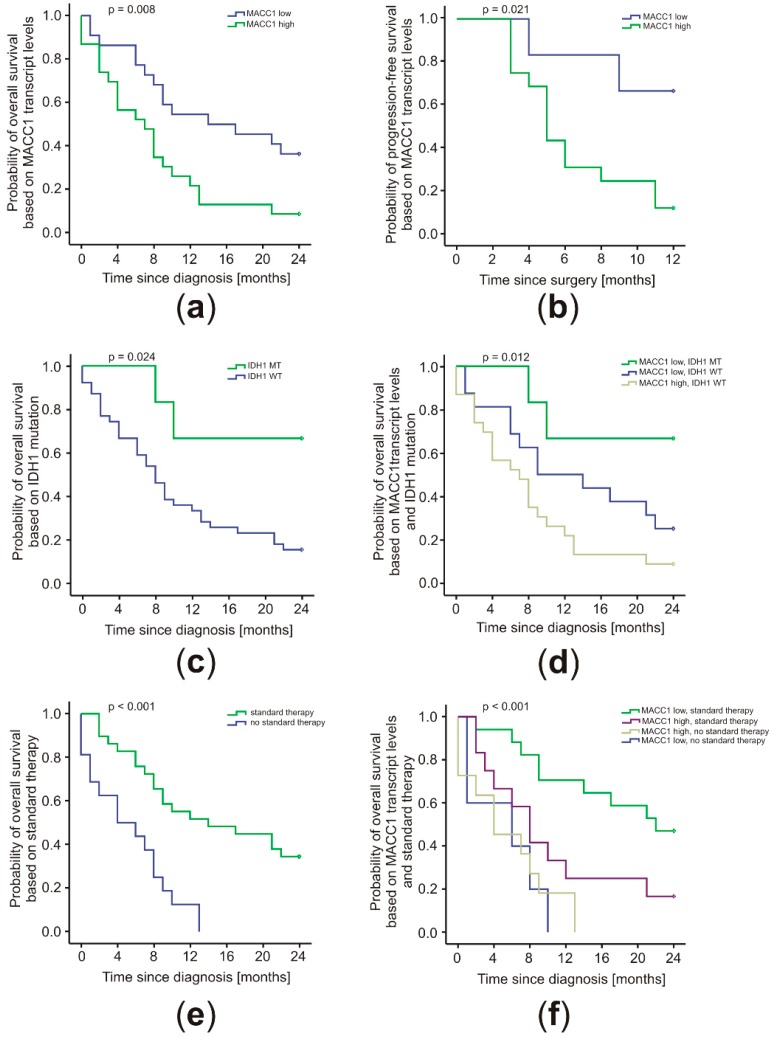
MACC1 levels correlated with patient prognosis conjoined with the IDH1 mutation status and treatment regimen. The cut-off values to distinguish low and high plasma levels of MACC1 were determined by receiver–operator characteristics (ROC) analyses and patient survival was visualized by Kaplan–Meier plots. (**a**) 24 months OS, cut-off = 0.384% calibrator; MACC1 low *n* = 22; MACC1 high *n* = 23) and (**b**) 12 months progression-free survival (PFS) (cut-off = 0.216% calibrator; MACC1 low *n* = 7; MACC1 high *n* = 15) of all GBM patients. (**c**) 24 months OS of GBM patients based on the status of IDH1 R132H mutation (IDH1 MT *n* = 5; IDH1 WT *n* = 36) in the tumor and (**d**) in combination with MACC1 transcript level (MACC1 low, IDH1 MT *n* = 5; MACC1 low, IDH1 WT *n* = 14; MACC1 high, IDH1 WT *n* = 17). (**e**) 24-month OS of patients receiving the standard therapy regimen (operation and radiochemotherapy according to Stupp [1]) compared to the OS of patients without standard therapy treatment (standard therapy *n* = 29; no standard therapy *n* = 16) and (**f**) in combination with the MACC1 level (MACC1 low, standard therapy *n* = 17; MACC1 high, standard therapy *n* = 12; MACC1 low, no standard therapy *n* = 5; MACC1 high, no standard therapy *n* = 11). Statistical analysis of patient survival was performed via log-rank tests.

The clinical course of the 45 GBM patients was followed for 24 months. Cut-off values for Kaplan–Meier analyses were determined by receiver–operator characteristics (ROC) calculations. Without considering different treatment regimens, high MACC1 mRNA levels in patient plasma were found to be prognostic for the 24 months OS (*p* = 0.008; cut-off = 0.384% calibrator) (Figure 2a) and 12 months PFS after diagnosis (*p* = 0.021; cut-off = 0.216% calibrator) (Figure 2b). The median OS of 14.5 months (SD = 7.2; 95% CI 0.4–28.6) for patients with low levels of MACC1 mRNA in their blood was higher than the 8.1 months (SD = 1.9; 95% CI 4.3–11.9) seen for patients with high MACC1 mRNA levels. The 24-month survival rate was 36% (SD = 10) and 9% (SD = 6), respectively. Patients with low levels of MACC1 did not reach a median PFS after 12 months. Their mean PFS was 9.7 months (SD = 1.2; 95% CI 7.3–12.0). In contrast, patients with high MACC1 mRNA levels had considerably lower median PFS of 5.4 months (SD = 0.4; 95% CI 4.2–7.4) and mean PFS of 6.4 months (SD = 0.8; 95% CI 4.9–7.9).

Although a univariate Cox regression revealed that low MACC1 levels were beneficial for the patients (HR = 0.41, 95% CI 0.20–0.81; *p* = 0.011), a multivariate analysis showed a dependency on covariates (adjusted HR = 0.45, 95% CI 0.12–1.74, *p* = 0.249). Patients with IDH1 MT did not reach their median survival within 24 months. However, they had a higher mean survival of 19.1 months (SD = 2.9; 95% CI 13.5–24.7), compared to patients with IDH1 WT. The latter had a median survival of 8.2 months (SD = 0.8; 95% CI 6.5–9.8) and mean survival of 10.6 months (SD = 1.3; 95% CI 8.1–13.1) (Figure 2c). The 24-month survival rate for patients with IDH1 MT was 67% (SD = 19) and for patients with IDH1 WT was 15% (SD = 6), respectively. All patients with IDH1 MT also had low MACC1 levels. The survival data remained identical when both factors were analyzed together. These patients showed the most favorable outcome, and those with IDH1 WT and high MACC1 had the worst prognosis (median OS 8.1 months; SD = 1.9; 95% CI 4.3–11.9). Patients with IDH1 WT and low MACC1 were intermediate (median OS 9.1 months; SD = 5.5; 95% CI 0.0–19.8) (Figure 2d). The respective 24-month survival rates were calculated as 67% (SD = 19), 9% (SD = 6), and 25% (SD = 11). No patients in this cohort displayed an IDH1 MT and high MACC1 expression (Figure 2d).

Patients receiving the standard therapy comprising operation and radiochemotherapy according to Stupp [1] benefited with a median OS of 14.5 months (SD = 6.6; 95% CI 1.6–27.4), compared to patients not receiving the standard regimen with a median OS of 4.4 months (*p* < 0.001; SD = 2.5; 95% CI 0.0–9.3) (Figure 2e). The 24-month survival rate of patients receiving radiochemotherapy with TMZ and adjuvant TMZ was 35% (SD = 9%), whereas all patients without standard treatment died within 13 months (Figure 2e). Importantly, MACC1 expression levels were associated with the therapy response of GBM patients (*p* < 0.001) (Figure 2f). Patients with low MACC1 levels receiving the standard therapy survived longest (median OS 22.6 months). Patients with high MACC1 showed shorter median OS of 8.1 months (SD = 0.8; 95% CI 6.5–9.7). The 24 months survival rate for patients with low MACC1 was higher (47%; SD = 12) compared to those patients with high MACC1 (17%; SD = 11). Patients not receiving the standard regimen showed the worst outcome, independent of their MACC1 levels (MACC1 low: 6.8 months SD = 6.0; 95% CI 0.0–18.6; MACC1 high: 4.4 months SD = 3.0; 95% CI 0.0–10.3).

## 3. Discussion

Circulating blood-based biomarkers are considered to be of great need for diagnosis, molecular characterization, and treatment response determination for gliomas. Usability of circulating tumor cells, circulating DNA, circulating micro RNAs, and circulating extracellular vesicles are currently under promising evaluation [9]. Recently, we found that MACC1 is overexpressed in GBM and associated with diminished patient survival [16]. Circulating MACC1 transcripts have been found in the peripheral blood of cancer patients and have been established as prognostic plasma marker for several solid cancer entities [13,14,15]. Our data now show that MACC1 transcripts can also be detected by liquid biopsies in GBM patients and that their level is of prognostic value. Low MACC1 plasma levels coincided with better outcome for patients with GBM. Although not an independent prognostic factor, cluster analysis revealed that low MACC1 levels clustered together with other favorable markers, especially with patients harboring IDH1 R132H mutations in their tumors. IDH1 belongs to the group of isocitrate dehydrogenase enzymes, which are metabolic enzymes of the citric acid cycle catalyzing the decarboxylation of isocitrate to α-ketoglutarate [17]. Characteristic mutations of IDH1/IDH2 are found in about 10% of all GBM, and the IDH mutation status has been included into the new WHO classification of GBM in 2016 [6,7]. These GBM are, for the most part, the former secondary GBM. The majority of IDH mutations in GBM represent the replacement of arginine 132 by histidine in IDH1 [17], resulting in the loss of native enzymatic activity [18] as well as gain of the ability to produce 2-hydroxyglutarate [19]. These enzymatic changes evoke a DNA hypermethylation phenotype, which are epigenetic alterations leading to a deregulation of gene activity [20]. GBM with wildtype IDH is the most common and aggressive form and was formerly largely classified as primary GBM [21]. In contrast to IDH WT GBM, patients with IDH1 R132H mutation have an improved prognosis [22]. Despite the small sample size, we noticed that low MACC1 plasma levels were associated with IDH1 R132H mutations. We did not find cases of high MACC1 expression associated with this mutation in our cohort. MACC1 has been discovered as metastasis-associated expressed protein in colon cancer [10]. Changes in DNA methylation play a role in the pathogenesis of colorectal cancer [23]. Although direct regulation of MACC1 by altered promoter-methylation has not yet been described [23], expression of at least two of its regulators, the micro RNAs miR-218 and miR-338-3p, is regulated by methylation of their genes [24,25]. miR-338-3p has been shown to regulate MACC1 expression in GBM cells [26]. Therefore, we plan to examine whether the hypermethylation phenotype of IDH1 R132H mutated GBM might be directly involved in downregulation of MACC1 expression in the future.

In accordance with previous publications [27,28], patients with IDH1 MT GBM had a better outcome in comparison to those with IDH1 WT. MACC1 levels were associated with the patients’ prognosis in conjunction with the IDH mutation status and treatment regimen. In an analysis of a prospectively collected molecular registry of 274 Chinese GBM patients, it was reported that patients with mutated IDH1 and methylated MGMT gene promoter had the best outcome. Meanwhile, those with IDH1 WT and unmethylated MGMT were poorest and those with either IDH1 MT or methylated MGMT exhibited intermediate survival [28]. These data were confirmed in several other studies of GBM patients receiving concurrent TMZ-based radiochemotherapy [29,30]. While in our analysis both clusters were equal in their MGMT methylation status, we received a very similar result when we based the Kaplan–Meier survival analysis on MACC1 expression level and IDH1 mutation status. Patients with low MACC1 levels and IDH1 MT survived longest. Those with high MACC1 levels and IDH1 WT, on the other hand, had the most unfavorable prognosis, while those with IDH1 WT but low MACC1 transcripts in their plasma were intermediate. This highlights the importance of MACC1 as additional and pre-operatively detectable prognostic markers further underlined by the fact that the MACC1 level was highly interrelated to the treatment response of the patients. Those patients with low MACC1 transcripts detectable and receiving the standard treatment regimen had the best prognosis, while those patients treated according to the standard regimen but with high MACC1 levels did nearly as bad as those patients not receiving the standard of care, irrespective of their MACC1 expression levels.

MACC1 is a transcriptional regulator of the receptor tyrosine kinase MET. MACC1 induced activation of the HGF/MET signaling pathway results in enhanced cell motility, invasion, and metastasis [10,11,31]. We investigated the effect of MACC1 overexpression in GBM cells and in murine organotypic hippocampal slice cultures. The proliferative, migratory, and tumor-formation abilities of GBM cells were significantly boosted by MACC1 expression [16]. MACC1 silencing in human U251 GBM cells by siRNA resulted in inhibition of cell proliferation, invasion, and migration, as well as increased apoptosis [32]. These cells were sensitized towards Cisplatin compared to normal U251 cells [33]. Recently, it was shown that miRNA-598 serves tumor-suppressive roles in GBM cell lines and that these effects are mediated through direct suppression of MACC1 expression [34].

So far, the only marker directly connected to treatment response of GBM patients is the MGMT promoter-methylation status [5]. Gene inactivating methylation is present in about 40% of GBM. This causes attenuated levels of this DNA repair enzyme sensitizing tumor cells to alkylating drugs [35]. O^6^-benzylguanine has been tested as an inhibitor of MGMT-activity in clinical trials. The outcome was disappointing due to high toxicity and lack of benefit for the patients [36]. MGMT promoter-methylation cannot be determined by liquid biopsy, as it is possible for the presence of circulating MACC1 transcripts and its promoter-methylation cannot be therapeutically influenced yet. Different strategies to suppress the expression or activity of mutated IDH1 or to inhibit the production of 2-hydroxyglutarate with metabolites such as oxaloacetate have been evaluated in vitro and in clinical trials including a currently performed phase-I trial on gliomas [37]. There are, however, no strategies approved yet to therapeutically influence IDH1 expression or activity for GBM treatment. The level of MACC1 expression is not only of prognostic relevance but can be therapeutically manipulated [11]. The tumor-derived cytokine endothelial-monocyte-activating polypeptide-II (EMAP-II) weakens the blood–tumor barrier, has antitumor activity, and, in combination with TMZ, suppresses the malignant behavior of human GBM stem cells in vitro and in vivo [38,39,40]. EMAP-II increases the expression of miR-590-3p, which in turn downregulates MACC1 [41]. Another potential therapeutic is the chimeric antibody Chanti-MACC1, which targets MACC1 directly and inhibits proliferation, migration, and invasion of cancer cells. In a mouse model, Chanti-MACC1 leads to diminished tumor growth, reduced metastasis, and promotes the long-term survival of the animals [42]. Most promising is the application of statins, a widely known and clinically used drug class for reducing cholesterol levels [43]. Lovastatin and Rottlerin emerged as the most potent MACC1 transcriptional inhibitors in a luciferase reporter-based high-throughput screening of more than 30,000 compounds of the ChemBioNet library. Lovastatin impairs MACC1 promoter activity, thereby inhibiting MACC1 transcription and limiting metastatic spread in preclinical mouse models [44]. It is able to pass the blood–brain barrier [45] and was the first FDA-approved cholesterol-lowering drug [46]. In the past years, the inhibitory activity of statins towards GBM was shown [47,48,49,50]. Statins have a good safety profile in general, including GBM patients [51]. They are applied daily for long time periods for the treatment of hypercholesterolemia. Analyzing a larger cohort of more than 300 GBM patients, Gaist et al. have shown a beneficial effect of prediagnostic statin use [52]. Our study now offers a non-invasive selection option to identify GBM patients with a molecular basis for statin treatment. Therefore, the translation from bench to bedside to sensitize GBM patients for therapy response by MACC1 inhibition should be straightforward.

## 4. Materials and Methods 

### 4.1. Standard Protocol Approvals and Patient Consents

Informed written consent of patients was obtained for this study in accordance with the International Conference on Harmonization, the declaration of Helsinki, as approved by the Institutional Review Board of the University of Würzburg (# 135/09).

### 4.2. Plasma Samples

From April 2012 until March 2014, preoperative blood specimens from 54 patients who underwent surgery for a suspected GBM were collected in the Department of Neurosurgery at the University Hospital Würzburg, Germany. The brain tumors were classified by routine histology based on WHO criteria [53] and re-evaluated after the revision of these criteria had been published [6]. Nine samples had to be excluded, because surgical or histological findings revealed a different etiology (WHO grade II-III glioma, metastases, inflammatory lesions) or the patients died within 4 weeks after surgery for reasons not related to the tumor disease. In total, 45 patients with a confirmed GBM remained evaluable and their clinical course was followed for 24 months (Table 1). Due to lack of sufficient tissue samples, the MGMT promoter methylation status could not be evaluated for 5 cases. Blood samples from 15 healthy donors served as control.

The tumor volumes at diagnosis were measured using T1-weighted MPRage postcontrast MRIs (MAGNETOM Trio; Siemens, Erlangen, Germany) with DISPImage. Regions of interest (ROI) were created manually on every DICOM-image slide to calculate the volume of interest based on ROIs and slice thickness.

Previously, we determined optimal conditions for blood taking, storage, and plasma separation [14]. MACC1 levels were not altered when plasma separation was carried out during the first 24 hours (cooled or kept at room temperature). Plasma was generated from cooled EDTA blood on the same day within 7 hours post blood taking. The procedure for plasma separation was as previously described [54,55]. Further, 5 ml of cooled EDTA-treated blood was centrifuged at 1300 rpm for 10 min at 10 °C. The plasma supernatant was again centrifuged at 2500 rpm for 15 min at 4 °C to remove all cell debris. Samples were stored at −80 °C as 400 µL aliquots. Investigators were blinded to the design, so that neither prognostic markers, nor the clinical course of the corresponding patient was disclosed during PCR-analysis.

### 4.3. Quantitative RT-PCR

Isolation of total RNA from plasma samples was performed using the high pure viral RNA kit (Roche Diagnostics, Mannheim, Germany) following the manufacturer instructions [55]. qRT-PCR was performed with some modifications as previously described [13,14,55]. MACC1 expression analysis was performed based on the hybridization probe detection format using amplicon-specific hybridization probes with the LightCycler 480 system (Roche Diagnostics, Mannheim, Germany). Briefly, after 10 min at 95 °C, we run 45 cycles each built of 10 s at 95 °C, 30 s at 60 °C, 4 s at 72 °C, followed by melting curve analysis (40 °C to 95 °C) after the PCR cycles (DNA Master HybProbe kit, Roche Diagnostics, Mannheim, Germany). A 136 bp MACC1-specific PCR product was amplified with the following primers and probes: Forward primer 5′-TTCTTTTGATTCCTCCGGTGA-3′, reverse primer 5′-ACTCTGATGGGCATGTGCTG-3′, FITC-probe 5′-GCAGACTTCCTCAAGAAATTCTGGAAGATCTA-3′, LCRed640-probe 5′-AGTGTTTCAGAACTTCTGGACATTTTAGACGA-3′ (primers: BioTeZ, probes: TIB MolBiol, Berlin, Germany). MACC1 mRNA expressions are given as percentage of the mRNA expression of a calibrator sample. The calibrator cDNA derived from the cell line SW620 (authentication by short tandem repeat genotyping, DSMZ, Braunschweig, Germany). This calibrator RNA was used in serial dilutions for generating a standard curve simultaneously in each quantitative PCR run. The in-run standard curve ranged from 100% calibrator down to 0.05% calibrator sample. Each sample was run and calculated in duplicate, and the means are depicted.

### 4.4. Immunohistochemistry (IHC) and Methylation-Specific High-Resolution Melting (HRM) Analysis

IHC was performed to evaluate the IDH1 R132H mutation status and HRM to determine the MGMT promoter-methylation status of the tumor samples as described elsewhere [56]. 

### 4.5. Statistics

Boxplots were compared using the Mann–Whitney U Test for independent samples of two groups or ANOVA for independent samples of more than two groups. Survival rates were calculated with Kaplan–Meier estimator analysis. Cut-offs in expression values of circulating MACC1 transcript levels were determined using receiver–operator characteristics (ROC) analysis and taking the value with the highest Youden index. Differences in survival rates were assessed using the log-rank test. Clustering of patients on the basis of the indicated parameters was performed by two-step cluster analysis, using log likelihood as a distance measure and Schwarz’s Bayesian (BIC) as cluster criterion. *p* ≤ 0.05 was considered statistically significant. Univariate and multivariate analyses were performed by application of the Cox proportional hazards model. All computations were made using IBM SPSS Statistics Version 21.

## 5. Conclusions

We identified MACC1 as an additional new prognostic marker for GBM patients [16], which can be determined pre-operatively by liquid biopsy and whose addition to the existing diagnostic workup may improve the accuracy of outcome prediction and, thus, help to define more precise risk categories of GBM patients. In addition, in the future therapeutically downregulating MACC1 may improve response of GBM patients to the current standard treatment regimen.

## Figures and Tables

**Table 1:** Clinical parameters of tumor samples.

**Patients’ characteristics**			
Sex	female: 11/24%		male: 34/76%
Median age at diagnosis	65 years		
ECOG at diagnosis	0: 16/36%	1: 21/47%	>1: 8/17%
**Tumor characteristics**			
Median tumor volume	36.0 cm^3^ (1.8–97.8 cm^3^)		
IDH1 R132H mutation	absent: 39/87%		present: 6/13%
MGMT promoter methylation ^1^	unmethylated: 20/50%		methylated: 20/50%
**Therapy**			
Radiation therapy	yes: 40/89%		no: 5/11%
Chemotherapy with TMZ	yes: 29/64%		no: 16/36%
**Outcome**			
OS	0–6 m: 16/36%		>6 m: 29/64%
PFS ^2^	0–6 m: 13/57%		>6 m: 10/43%

Given are the absolute numbers of patients and the percentages of the analyzed population. ^1^ Due to lack of sufficient tissue samples, the MGMT promoter methylation status could not be re-evaluated for some patients. ^2^ Some patients were subtotally resected or biopsied and therefore, the PFS could not be determined. ECOG = Eastern Cooperative Oncology Group score; OS = overall survival; PFS = progression free survival; m = months; TMZ = temozolomide.

**Table 2:** Cluster analyses.

	**Patients**			**Female**		**Male**		Age *		**OS**	
	n	%		n	%	n	%	years	SD	months/days	SD
Cluster 1	18	46		3	30	15	52	57.0	11.9	16.0/488	8.5/259
Cluster 2	21	54		7	70	14	48	69.1	9.3	9.3/283	6.9/209
Combined	39	100		10	100	29	100	63.5	12.1	12.4/377	8.3/252
	**MACC1 status**					MACC1 *			**MGMT status**		
	low		high			%calibrator		SD	not methylated		methylated
Cluster 1	18		0			0.20		0.10	9		9
Cluster 2	0		21			0.84		0.41	11		10
Combined	18		21			0.54		0.44	20		19
	**IDH1 R132H mutation**							**tumor volume**			
	absent					present		cm^3^			SD
Cluster 1	14					4		33			24
Cluster 2	21					0		44			28
Combined	35					4		39			26

Centroids and standard deviations (SD) of patient parameters after clustering. * *p* < 0.05. OS = overall survival.
